# Effects of Addition of Lanthanum and Zinc Oxides on the Biological Properties of TiO_2_–SiO_2_–P_2_O_5_/CaO on Ion Exchange Resin for Bone Implantation

**DOI:** 10.1021/acsomega.3c08268

**Published:** 2024-02-02

**Authors:** Ekaterina S. Lyutova, Valeriya A. Tkachuk, Alexandra M. Zakharkiva, Lyudmila P. Borilo, Aleksandr A. Buzaev, Yu-Wen Chen

**Affiliations:** † National Research Tomsk State University, 36 Lenina Avenue, Tomsk 634050, Russia; ‡ Department of Chemical Engineering, National central University, Jhongli 32001, Taiwan

## Abstract

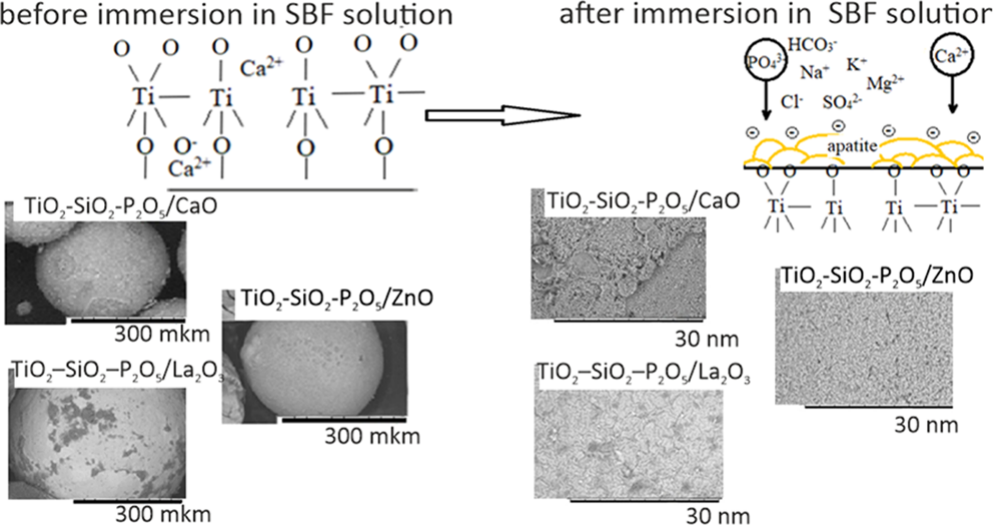

The spherical materials TiO_2_–SiO_2_–P_2_O_5_/CaO, TiO_2_–SiO_2_–P_2_O_5_/La_2_O_3_, and TiO_2_–SiO_2_–P_2_O_5_/ZnO deposited on the Tokem-250 cation exchanger have been synthesized with an alcoholic solution by the sol–gel method. The macroporous cation exchanger Tokem-250, which has high Ca^2+^, Zn^2+^, and La^3+^ ion selectivity, was used in the present study. This material has the ability to precipitate and mineralize calcium phosphates on its surface in biological media, since it has high porosity, a homogeneous structure with a uniform variation of elements, and the presence of active centers (Si^4+^, Ti^4+^) on the surface. The effect of lanthanum and zinc additives on biological properties has been studied. It was established that accumulation of Ca^2+^ and Mg^2+^ occurs faster on the surface of TiO_2_–SiO_2_–P_2_O_5_/ZnO in the SBF (simulated body fluid) model solution, showing higher reaction capacity. The amount of calcium and phosphorus ions on the surface of sample TiO_2_–SiO_2_–P_2_O_5_/La_2_O_3_ is greater due to the ability of lanthanum to coordinate a large number of ions (lanthanum coordination number is 10). The presence of zinc ions in the system causes the partial hemoglobin release from erythrocytes into the supernatant fluid. The samples with lanthanum ions reduce the amount of protein in plasma after incubation, which has a positive effect on the practical application.

## 1 Introduction

Increasing interest in better quality of life and human lifespan is the main feature of the new millennium. Achieving this goal suggests, in particular, the creation of new materials for artificial organs and tissues.^1,2^

The human body has a limited ability to regenerate most parts of organs and tissues.^3−5^ In such circumstances, tissue engineering and regenerative medicine have a need for the development of new materials.^6,7^ Therefore, to address these issues, artificial bone implants have been developed.^8^ For clinical medicine, the biological activity and features of synthetic bone substitutes are of great importance.^9^ The main requirements for such materials are biocompatibility, bioactivity, and mechanical resistance.^10,11^ A principal application of bioactive materials is increasing the degree of local regeneration and simultaneously decreasing the probability of artificial material rejection.^12,13^ In this regard, the chosen materials should contain the elements that are part of the natural bone.^14−16^

Biopolymer- and bioceramic-based composite structures, composed of hydroxyapatite and collagen, which are the components of natural bones, have become a research topic in recent years. Calcium phosphates, due to their chemical affinity to the inorganic parts of solid biological tissues, are widely used in biomedical materials.^17^ These materials are similar in structure to the inorganic components of bones and teeth and are widely used in modern clinical practice. One of the promising materials is calcium phosphate coatings (based on CaO, SiO_2_, P_2_O_5_, and Na_2_O oxides). Such biomaterials are capable of bone fusion due to the formation of a calcium phosphate layer. The composition and structure of which are identical to the mineral composition of bones.^18−20^

In addition to the above oxides, other oxides can be a part of biomaterials, improving their characteristics, such as TiO_2_. The addition of TiO_2_ can stabilize the phosphate lattice of calcium phosphate materials. It has been reported that TiO_2_ can improve chemical stability and module of elasticity and increase the viscosity of calcium phosphate systems.^21−23^

The addition of other elements into the structure of the biomaterial can enhance the functional properties of the materials. To improve the characteristics (e.g., antiseptic properties) of the biomaterial, modification of calcium phosphate materials is needed. Adding zinc to the system can increase the stability of the biomaterial and prevent degrading, as well as give antioxidant and antibacterial properties to the material. Zinc ions in the structure of calcium phosphate materials initiate the growth of blood vessels, which has a positive effect on bone cells of living organism formation and metabolism.^24−26^ La_2_O_3_ can also be included in the composition of the biomaterial due to the large atomic radius, and the lanthanum atom has quite a large coordination number of 10. Therefore, the lanthanum atom can significantly easily attach to components needed for bone reformation in comparison with the calcium atom.^27,28^ The introduction of La_2_O_3_ in the structure of the biomaterial leads to high bending strength and a low dissolution rate.^29,30^ It was established that lanthanum ions have biological activity and antiseptic properties and also can affect various stages of the blood clotting process.^31^ However, lanthanum is a fairly toxic metal; therefore, it should be included in small amounts. Works^32^ in recent years have found that spherical composites TiO_2_–SiO_2_–P_2_O_5_/CaO based on Tokem-250 are promising biomaterials. When calcium ions are replaced partially by lanthanum or zinc ions in the structure of the material, the biological properties can be improved. The present work aimed to study the effect of adding lanthanum and zinc oxides on the biological properties of the materials of TiO_2_–SiO_2_–P_2_O_5_/CaO supported on the cation exchanger Tokem-250.

## 2 Experimental Section

### 2.1 Reagents

Tetraethoxysilane (Puriss. Spec., Germany), orthophosphoric acid (Puriss. spec. Himmed Russia), calcium nitrate (p. a. Himmed Russia), lanthanum nitrate (p. a. Himmed Russia), zinc nitrate (p. a. Himmed Russia), tetrabutoxytitanium (puriss. spec. Germany), and PVA-16/1 (JSC Nevinnomyssky Azot, Russia) were used in this study.

#### 2.1.1 Synthesis of Materials

**Figure 1:**
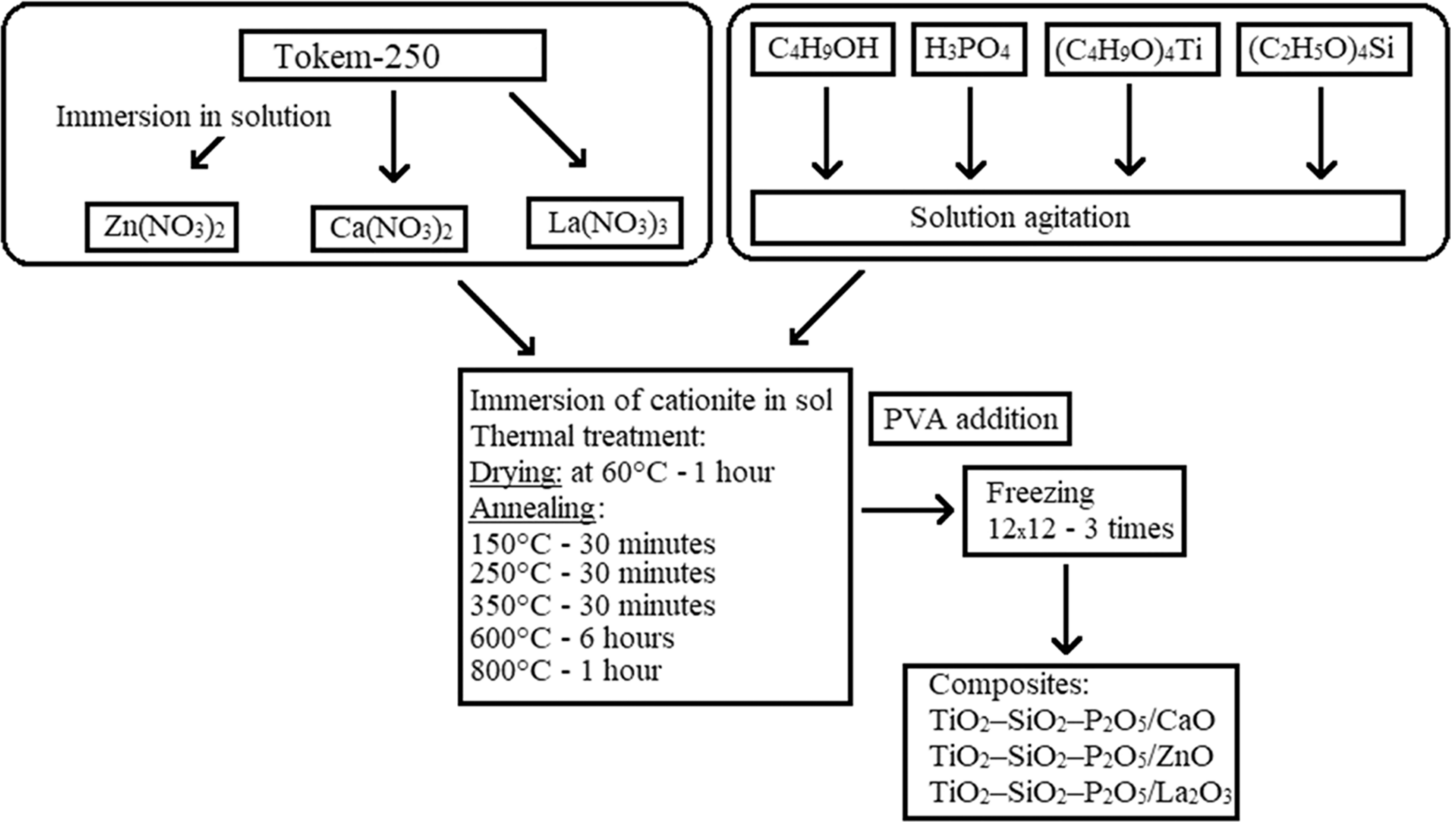
Scheme of material synthesis.

The as-synthesized materials should have the shape of spherical granules. To give the materials a spherical shape, the acryl-divinylbenzene cationite of Tokem-250 grade (NPO Tokem LLC, with an average grain size of 0.4–0.6 mm) was used as the framework. The inner part of the material was represented by calcium oxide in the material TiO_2_–SiO_2_–P_2_O_5_/CaO, lanthanum(III) oxide in the material TiO_2_–SiO_2_–P_2_O_5_/La_2_O_3_, and zinc oxide in the material TiO_2_–SiO_2_–P_2_O_5_/ZnO. Tokem-250 (weakly acidic porous cationite based on acryl-divinylbenzene) was chosen as a preform because of its high selectivity toward Ca^2+^, Zn^2+^, and La^3+^ ions. The top layer of the material is a film of TiO_2_–SiO_2_–P_2_O_5_ composition, with mass contents of oxides of 30, 65, and 3 wt %, respectively. The film was obtained by immersion of the cationite in the aggregated-stable sol for 1 day. The aggregated-stable sol was prepared according to the method described in the previous study.^32^ The samples were then dried at 60 °C for 1 h and subjected to stepwise heat treatment according to the scheme presented in the paper.^32^ The binder additive PVA was added to the tested samples at a ratio of 1:1, followed by triple freezing for 12/12 h (Figure 1).

### 2.2 Characterization Methods

The biological properties of the materials were investigated using the technique proposed by Kokubo in the modeling fluid (SBF) solution.^33^ The spherical materials were incubated in the SBF solution at 37 °C for 14 days with daily renewal of the solution. The concentration of calcium and magnesium ions in the solution after immersion was determined by trigonometric titration. The ion accumulation coefficient on the surface was calculated by the following formula , where ΔC (Ca^2+^, Mg^2+^) is the total change in concentration during time interval τ (in days).^34^

The acid–base properties of the solid body surface, which were formed during synthesis and reflect the features of its structure and reacting capacity, were investigated by the method of pH-metry, which was based on the change in the pH value of the aqueous suspension in time.

The infrared spectra of the powders were obtained by using a Nicolet 6700 Fourier spectrometer (Thermo Scientific) in the 400–4000 cm^–1^ range.

The structure and chemical composition of the samples were investigated by scanning electron microscopy and energy-dispersive X-ray spectroscopy using a Hitachi TM-3000 electron microscope (Thermo Fisher Scientific) with a ShiftED 3000 attachment for micro X-ray spectral analysis.

The adsorption of plasma proteins was studied by a modified solution absorption method. This method represents two quantitative determinations of protein concentration in blood plasma before and after the incubation of samples.^35^ Plasma was isolated from the whole hemostatic blood of a healthy donor by a centrifugation method. After that, the protein content was determined by the biuret reaction method. The samples were poured into 2 mL of plasma and incubated in the thermostat at 37 °C for 24 h. After incubation, a similar determination of the protein concentration was performed. The difference between the protein concentration in intact plasma and after the incubation of samples was used to obtain the value of plasma protein adsorption.

Hemostate blood from a healthy donor was used to assess the hemocompatibility of the samples. The blood was centrifuged and separated erythromas. The resulting erythromas was diluted with a sterile 1X PBS solution at 37 °C in a 1:9 ratio. The samples were placed in a standard 24-well cell culture plate and filled with the obtained blood solution in PBS at a ratio of 1 mL of solution per 1 cm^2^ of sample surface area. Deionized water was used as a negative control, and 1× PBS solution was used as a positive control. The hemolysis level of the negative control was taken as 100%, and the hemolysis level of the positive control was taken as 0%. Afterward, the plate was incubated in a thermostat at 37 °C for 60 min.

After that, blood from the wells of the plate was transferred to centrifuge tubes and centrifuged for 5 min at 3000 rpm to precipitate the remaining erythrocytes. The supernatant was then carefully removed and transferred to a standard 96-well plate for spectroscopic analysis at 545 nm using a Tecan Infinite F50 ELISA reader (Tecan Inc.). The percentage of hemolysis was calculated using the following formula.
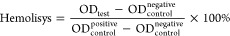
1

## 3 Results and Discussion

**Figure 2:**
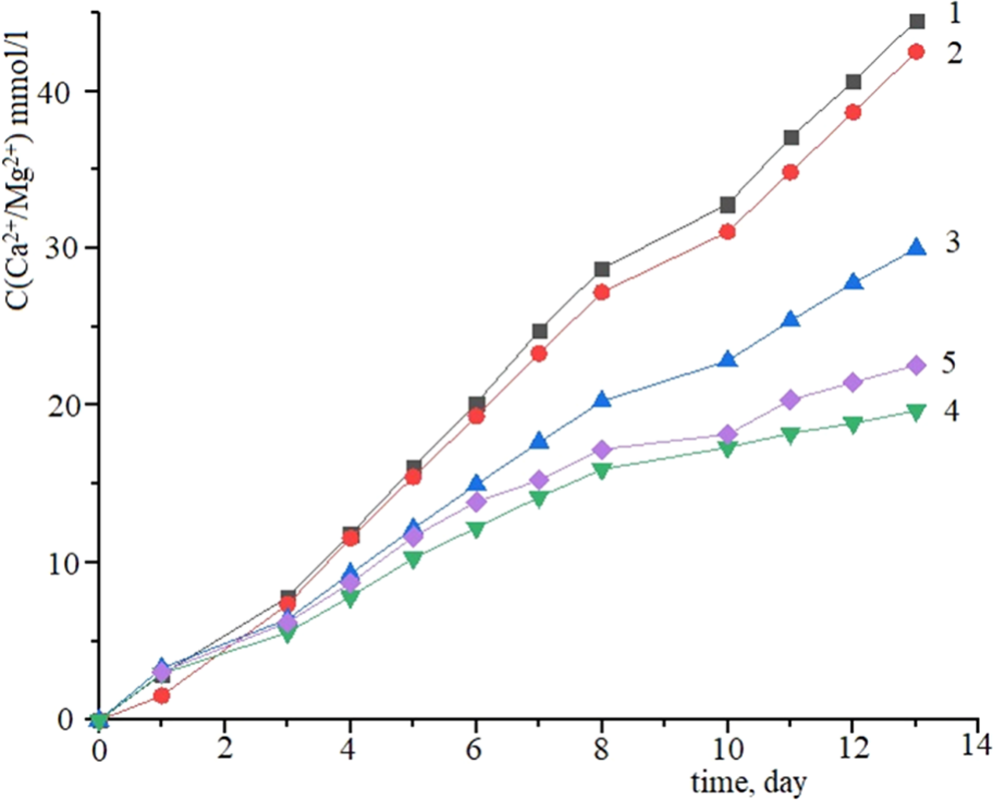
Accumulation curves of Ca^2+^ and Mg^2+^ ions versus time in the SBF solution: 1—TiO_2_–SiO_2_–P_2_O_5_/CaO, 2—TiO_2_–SiO_2_–P_2_O_5_/ZnO, 3—TiO_2_–SiO_2_–P_2_O_5_/Ca:TiO_2_–SiO_2_–P_2_O_5_/ZnO ratio = 1:1, 4—TiO_2_–SiO_2_–P_2_O_5_/La_2_O_3_, and 5—TiO_2_–SiO_2_–P_2_O_5_/CaO:TiO_2_–SiO_2_–P_2_O_5_/La_2_O_3_ ratio = 1:1.

To establish the influence of zinc and lanthanum oxides on the ability of the materials to form a calcium phosphate layer on the surface, five samples were immersed in the model SBF (simulated body fluid) solution: 1—TiO_2_–SiO_2_–P_2_O_5_/CaO, 2—TiO_2_–SiO_2_–P_2_O_5_/ZnO, 3—TiO_2_–SiO_2_–P_2_O_5_/CaO:TiO_2_–SiO_2_–P_2_O_5_/ZnO ratio = 1:1, 4—TiO_2_–SiO_2_–P_2_O_5_/La_2_O_3_, and 5—TiO_2_–SiO_2_–P_2_O_5_/CaO:TiO_2_–SiO_2_–P_2_O_5_/La_2_O_3_ ratio = 1:1. Figure 2 shows the accumulation curves of Ca^2+^ and Mg^2+^ ions in the SBF solution. The graph shows that the ion precipitation occurred in three stages; for each stage, the ion accumulation coefficient on the surface of the material was calculated and is listed in Table 1.

**Table 1:** Accumulation Coefficient of Ca^2+^ and Mg^2+^ Ions in the Samples in the SBF Solution

sample	*k* (0–3 days)	*k* (4–8 days)	*k* (9–14 days)
TiO_2_–SiO_2_–P_2_O_5_/CaO	1.62	1.28	0.87
TiO_2_–SiO_2_–P_2_O_5_/ZnO	1.78	1.73	1.22
TiO_2_–SiO_2_–P_2_O_5_/CaO:TiO_2_–SiO_2_–P_2_O_5_/ZnO = 1:1	1.48	1.66	1.21
TiO_2_–SiO_2_–P_2_O_5_/La_2_O_3_	1.12	1.22	0.75
TiO_2_–SiO_2_–P_2_O_5_/CaO:TiO_2_–SiO_2_–P_2_O_5_/La_2_O_3_ = 1:1.	1.30	1.02	0.50

In the first 3 days, precipitation occurred at a high rate (accumulation coefficient has values in the range of 1.48–1.78), migration of alkali and alkaline-earth ions at the sample–solution interface proceeds, interaction with active centers, which are titanium and silicon atoms, on the surface of the material also proceed. The precipitation rate is higher for the sample TiO_2_–SiO_2_–P_2_O_5_/CaO. This can be explained by the fact that calcium ions present in the sample can also act as active centers. However, the presence of lanthanum in the samples reduces the precipitation rate of ions due to the larger radius and lower activity of lanthanum.

In the second stage, as Ca^2+^ and Mg^2+^ ions accumulated and an amorphous layer was formed on the surface of the samples, the precipitation rate decreased. In the third stage (after 9 days), the stabilization of the ion precipitation on the surface of samples (accumulation coefficient in the range of 0.5–1.22) occurred. It was found that the accumulation of Ca^2+^ and Mg^2+^ ions occurred faster in the TiO_2_–SiO_2_–P_2_O_5_/ZnO sample, which indicates a higher activity of the formation of a calcium phosphate layer on the material surface in the SBF solution.

**Figure 3:**
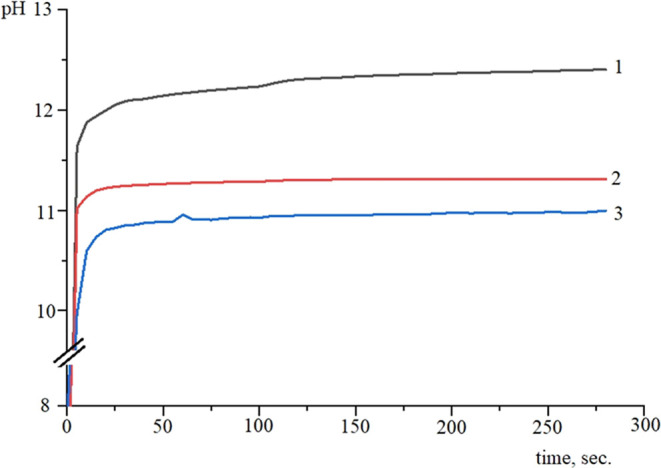
Kinetic curves of the acidity (pH) change in aqueous suspensions: 1—TiO_2_–SiO_2_–P_2_O_5_/CaO, 2—TiO_2_–SiO_2_–P_2_O_5_/ZnO, and 3—TiO_2_–SiO_2_–P_2_O_5_/La_2_O_3_.

The formation of an amorphous layer on the surface of the samples occurred due to the release of Na^+^ and Ca^2+^ ions from the surface layer by exchange with H_3_O^+^ ions in the liquid to form Ti–OH (Si–OH) groups. The Ti–OH (Si–OH) groups were formed and then dissociated into Ti–O– (Si–O–) ions, which interacted with positively charged Ca^2+^ ions. As calcium ions accumulated on the surface, the surface gradually acquired an overall positive charge. As a result, the positively charged surface combined with the negatively charged phosphate ions, resulting in the formation of amorphous calcium phosphate.^36,37^ Therefore, to form an amorphous layer in biological fluids, the surface of the material should have a negative charge. In this work, the acid–base properties of the solid surface were evaluated by pH-metry. Kinetic curves of the acidity change (pH) of the aqueous suspensions of the samples are presented in Figure 3.

The surface charge affects the distribution of ions when the material is immersed in SBF. At the initial period of time, rapid alkalinization of the solution was observed. The high rate of pH value change indicates the presence of strong aprotonic centers of the basic type in aqueous suspensions.^38^

The subsequent course of the curves was characterized by insignificant changes in acidity. The plateau in the regime between 200 and 1200 s at sufficiently high pH values indicates the predominance of the strong proton centers of basic character on the surface.

**Figure 4:**
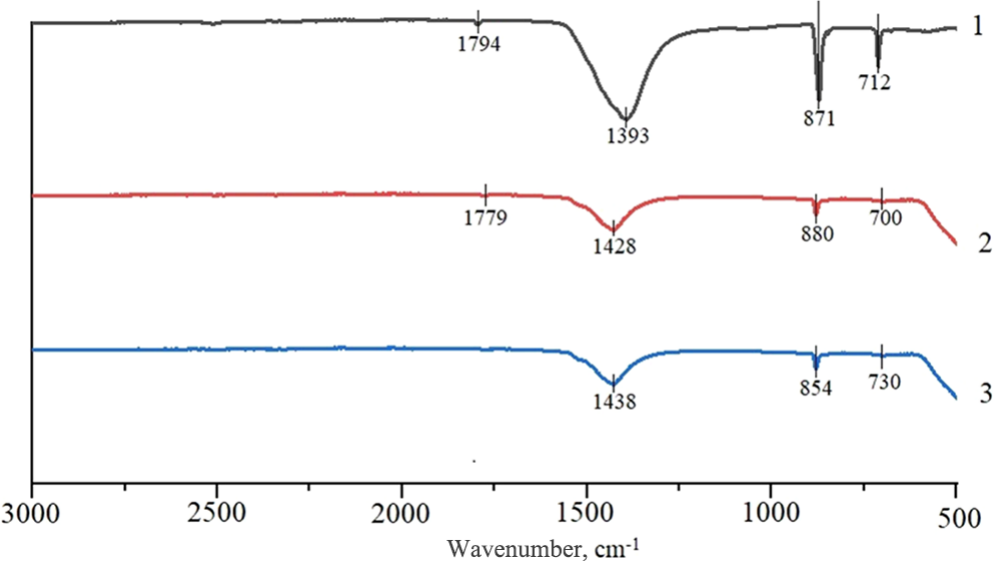
IR spectra of the studied samples:1—TiO_2_–SiO_2_–P_2_O_5_/CaO, 2—TiO_2_–SiO_2_–P_2_O_5_/ZnO, and 3—TiO_2_–SiO_2_–P_2_O_5_/La_2_O_3_.

**Figure 5:**
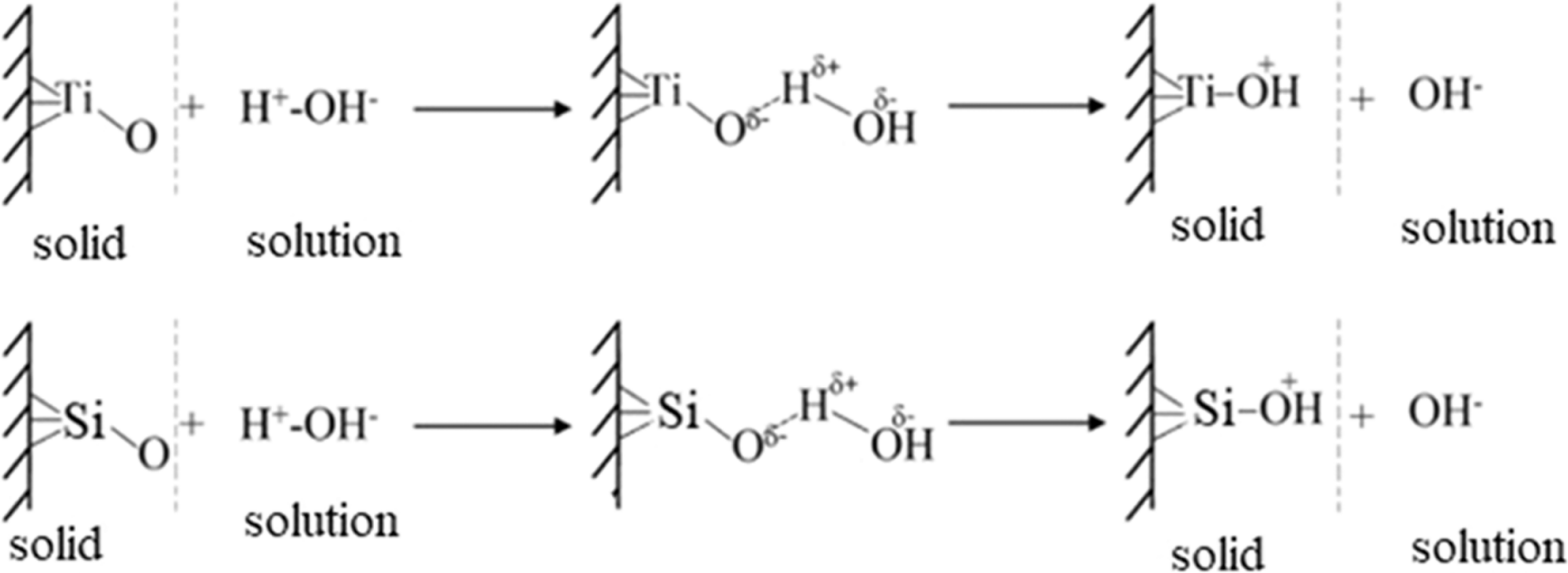
Mechanisms of hydroxyl ion formation on Lewis basic centers.

Thus, Lewis basic centers prevail on the surface of the samples. Since Si–OH and Ti–OH bonds were not identified in the samples by IR spectroscopy (Figure 4), it is possible to assume the formation of hydroxyl ions on the sample surface (Figure 5). This contributes to the formation of bonds with an apatite-like layer on the surface of the samples, which imitates natural bone tissue.

The structure of the materials was formed by silicon–oxygen and phosphorus–oxygen atomic groups. This is confirmed by the presence of bands at 859–880 cm^–1^ in the IR spectrum, which corresponds to the valence asymmetric vibrations of Si–O–Si, valence symmetric vibrations of Si–O–P, and valence symmetric vibrations of PO_4_^3–^. The valence symmetric vibrations of Si–O–Si and P–O–P bonds and strain vibrations of SiO_2_ and Ti–O are fixed. For the TiO_2_–SiO_2_–P_2_O_5_/La_2_O_3_ sample, the valence vibrations of the La–O bond were observed. The TiO_2_–SiO_2_–P_2_O_5_/ZnO sample shows an absorption band at 700 cm^–1^, which characterizes the presence of Zn–O–Ti bonds and a six-coordinated titanium atom TiO_6_.

The surface characteristics are important while studying the properties of biomaterials because they can influence the vital processes such as protein, cell adhesion, and bioresorbability of materials when implanted in the body. The bioactive properties depend on the charge and porosity of the material surface. The pore structure of all of the samples, S_sp_, was 110 m^2^/g, the total pore volume was 0.48 cm^3^/g, and the average pore size was 15–26 nm. The samples had high porosity, which is favorable for practical application.

**Figure 6:**
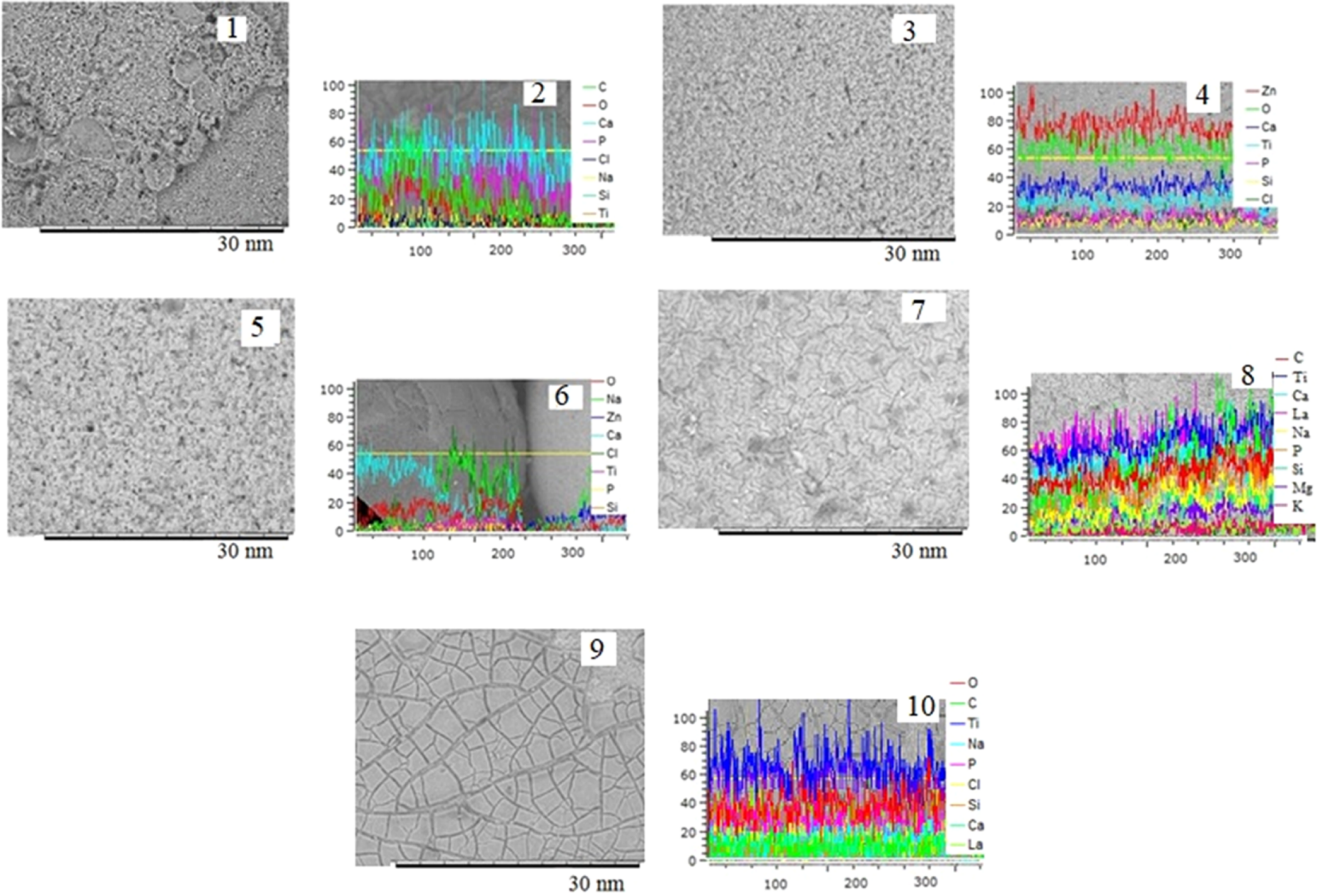
Microphotographs of the samples (1, 3, 5, 7, 9) and distribution of the elements along the line (2, 4, 6, 8, 10) after immersion in the SBF solution for 14 days: 1, 2—sample TiO_2_–SiO_2_–P_2_O_5_/CaO; 3, 4—sample TiO_2_–SiO_2_–P_2_O_5_/ZnO; 5, 6—sample with ratio TiO_2_–SiO_2_–P_2_O_5_/CaO:TiO_2_–SiO_2_–P_2_O_5_/ZnO = 1:1; 7, 8—sample TiO_2_–SiO_2_–P_2_O_5_/La_2_O_3_; and 9, 10—sample with the ratio TiO_2_–SiO_2_–P_2_O_5_/CaO_:_TiO_2_–SiO_2_–P_2_O_5_/La_2_O_3_ = 1:1.

The microphotographs and distributions of the elements on the surfaces of samples were obtained to compare the surface morphologies of the spherical composites before and after immersion in SBF solution (Figure 6). According to the results of X-ray spectral microanalysis (XRMS), the calcium, titanium, phosphorus, silicon, and lanthanum ions were precipitated on the surface. For the TiO_2_–SiO_2_–P_2_O_5_/La_2_O_3_ sample based on the Tokem-250 cationite, the highest amount of precipitated calcium and phosphorus ions was detected after immersion in the SBF solution. The contents of calcium ions increased by 9 times and phosphorus by 2 times. This is possible due to the structure of the lanthanum ion, which is characterized by a coordination number of 10. According to the results of IR spectroscopy, on the surface of the TiO_2_–SiO_2_–P_2_O_5_/La_2_O_3_ sample, the fixed valence vibrations of the La–O bond are present, which can act as the active centers.

When introducing composites into the biosphere, there is a need to bind spherical particles, and for this purpose, various binding additives can be used.^39,40^ In this work, poly(vinyl alcohol) (PVA), which is inert to the studied samples, was chosen as a binding additive. The samples were placed in the solution of poly(vinyl alcohol), and after three times 6 h freezing, it was immersed in SBF. It was found that PVA did not affect the precipitation of ions from SBF solution on the surface of the spherical TiO_2_–SiO_2_–P_2_O_5_/La_2_O_3_ composite on the cation exchanger Tokem-250.

To establish the influence of zinc/lanthanum oxide and the binder additive to form a calcium phosphate layer on the surface, six samples were immersed in SBF (simulated body fluid) model solution, i.e., 1—TiO_2_–SiO_2_–P_2_O_5_/CaO_PVA, 2—TiO_2_–SiO_2_–P_2_O_5_/ZnO_PVA, 3—TiO_2_–SiO_2_–P_2_O_5_/CaO:TiO_2_–SiO_2_–P_2_O_5_/ZnO_PVA ratio = 1:1, 4—Tokem-250_PVA, 5—TiO_2_–SiO_2_–P_2_O_5_/La_2_O_3_, and 6—TiO_2_–SiO_2_–P_2_O_5_/CaO:TiO_2_–SiO_2_–P_2_O_5_/La_2_O_3_ _PVA ratio = 1:1

**Figure 7:**
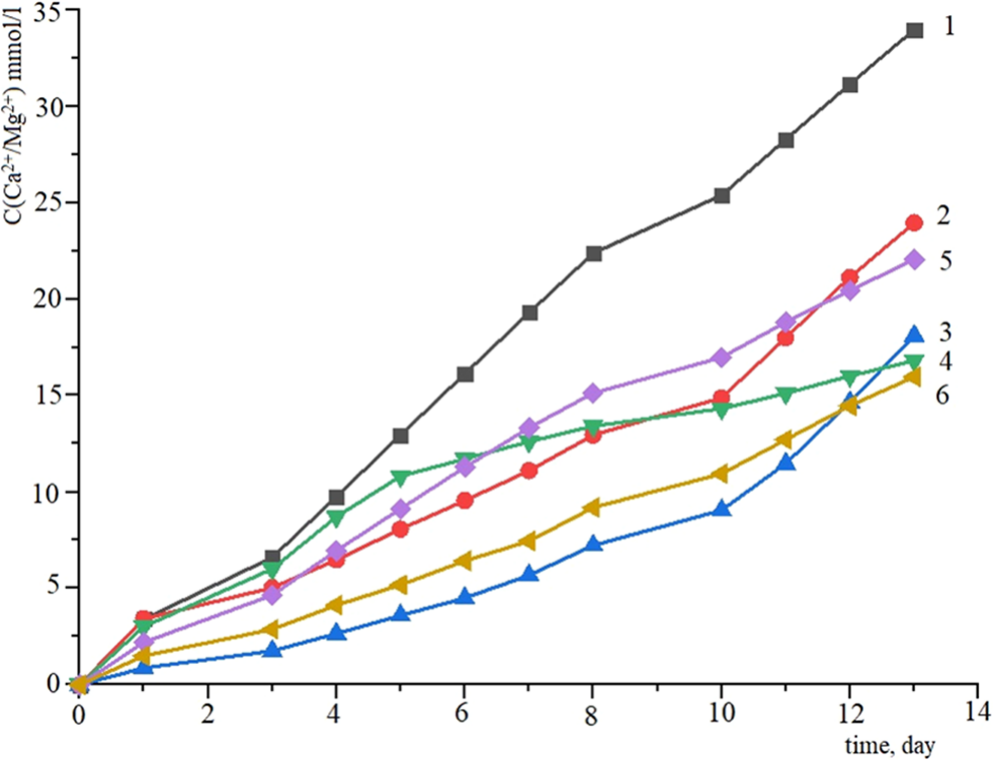
Accumulation curves of Ca^2+^ and Mg^2+^ ions versus time in the SBF solution: 1—TiO_2_–SiO_2_–P_2_O_5_/CaO_PVA, 2—TiO_2_–SiO_2_–P_2_O_5_/ZnO_PVA, 3—TiO_2_–SiO_2_–P_2_O_5_/CaO:TiO_2_–SiO_2_–P_2_O_5_/ZnO_PVA ratio = 1:1, 4—Tokem-250_PVA, 5—TiO_2_–SiO_2_–P_2_O_5_/La_2_O_3_, and 6—TiO_2_–SiO_2_–P_2_O_5_/CaO:TiO_2_–SiO_2_–P_2_O_5_/La_2_O_3_ _PVA ratio = 1:1.

Figure 7 shows the results of Ca^2+^ and Mg^2+^ ion accumulation in the SBF (simulated body fluid) solution. The graph shows that the precipitation of ions proceeds in three stages; for each stage, the ion accumulation coefficient on the surface of the material was calculated and is tabulated in Table 2.

**Table 2:** Accumulation Coefficient of Ca^2+^ and Mg^2+^ Ions of the Samples in SBF Solution

sample	*k* (0–3 days)	*k* (4–8 days)	*k* (9–14 days)
Tokem-250_PVA	1.20	0.16	0.41
TiO_2_–SiO_2_–P_2_O_5_/CaO_PVA	1.01	1.05	1.43
TiO_2_–SiO_2_–P_2_O_5_/ZnO_PVA	1.26	0.86	1.23
TiO_2_–SiO_2_–P_2_O_5_/CaO:TiO_2_–SiO_2_–P_2_O_5_/ZnO_PVA = 1:1	1.33	0.88	1.22
TiO_2_–SiO_2_–P_2_O_5_/La_2_O_3__PVA	1.02	0.75	0.99
TiO_2_–SiO_2_–P_2_O_5_/CaO:TiO_2_–SiO_2_–P_2_O_5_/La_2_O_3__PVA = 1:1.	1.09	0.98	1.01

The results show that the introduction of a binder additive slightly reduced the formation of an apatite-like layer on the surface of samples at the first stages. But after 9 days (stage 3), the accumulation coefficient was greater for all of the samples treated with PVA. In this work, the pH value of the solutions after immersion of the samples was measured. For all of the samples, the pH value increased up to 9. This indicates chemical processes on the surface of the materials when immersed in SBF solution. The increase in pH value creates a favorable environment for the formation of a calcium phosphate layer on the composite surface. It was found that the binder additive did not affect the stages and mechanism of formation of the calcium phosphate layer on the surface of the material. Thus, it was chosen to use PVA as a binder additive for further studies.

The ability to adsorb on the surface is an important factor for biomaterials because protein adsorption is the initial step that occurs on the implant surface after implantation. The proteins adsorbed on the surface contribute to the subsequent processes of adhesion, proliferation, and differentiation of the cells present in the local microenvironment around the implant.^41^ The high porosity of materials leads to the high adsorption and accumulation of various endogenous bone growth factors on the surface of the material.^41^

The ability to adsorb protein on the surface of the material was studied by a modified solution absorption method. This method represents two quantitative determinations of protein concentration in blood plasma before and after the incubation of samples (Table 3).

**Table 3:** Protein Contents in Plasma after Incubation and the Hemolysis Level

sample	protein content in the sample after incubation, g/mL	hemolysis level, %
Tokem-250_PVA	0.0456 ± 0.0088	0.0726 ± 0.4425
TiO_2_–SiO_2_–P_2_O_5_/CaO_PVA	0.0463 ± 0.0305	0.7178 ± 0.2323
TiO_2_–SiO_2_–P_2_O_5_/ZnO_PVA	0.0373 ± 0.011	28.0665 ± 0.6503
TiO_2_–SiO_2_–P_2_O_5_/CaO:TiO_2_–SiO_2_–P_2_O_5_/ZnO_PVA = 1:1	0.0401 ± 0.0031	18.1227 ± 0.2737
TiO_2_–SiO_2_–P_2_O_5_/La_2_O_3__PVA	0.0473 ± 0.001	1.9354 ± 0.2634
TiO_2_–SiO_2_–P_2_O_5_/La_2_O_3_:TiO_2_–SiO_2_–P_2_O_5_/CaO_PVA = 1:1	0.0421 ± 0.0021	1.3188 ± 0.2657

The experimental results (Table 3) show that for all samples, the amount of protein in plasma slightly decreased after incubation, which was confirmed statistically (*p* < 0.05). However, the TiO_2_–SiO_2_–P_2_O_5_/ZnO_PVA and TiO_2_–SiO_2_–P_2_O_5_/CaO:TiO_2_–SiO_2_–P_2_O_5_/ZnO_PVA = 1:1 samples had the greatest ability of protein adsorption because a significantly reduced protein concentration after incubation was found compared to intact plasma (*p* < 0.01).

The hemolytic indices for TiO_2_–SiO_2_–P_2_O_5_/ZnO_PVA and TiO_2_–SiO_2_–P_2_O_5_/CaO:TiO_2_–SiO_2_–P_2_O_5_/ZnO_PVA = 1:1 samples were found to be 28.0665 ± 0.6503% and 18.1227 ± 0.2737%, respectively, representing the highest hemolysis levels in these samples (Table 3). Meanwhile, the hemolysis levels of the other samples did not differ from the positive control (*p* > 0.05). Also, there are no statistically significant differences among samples TiO_2_–SiO_2_–P_2_O_5_/CaO_PVA, Tokem-250_PVA, TiO_2_–SiO_2_–P_2_O_5_/La_2_O_3__PVA, TiO_2_–SiO_2_–P_2_O_5_/La_2_O_3_, and TiO_2_–SiO_2_–P_2_O_5_/CaO_PVA = 1:1.

The color intensity of the supernatant in samples TiO_2_–SiO_2_–P_2_O_5_/ZnO_PVA and TiO_2_–SiO_2_–P_2_O_5_/CaO_PVA:TiO_2_–SiO_2_–P_2_O_5_/ZnO_PVA = 1:1 significantly exceeded the level of the positive control. Consequently, samples TiO_2_–SiO_2_–P_2_O_5_/ZnO_PVA and TiO_2_–SiO_2_–P_2_O_5_/CaO:TiO_2_–SiO_2_–P_2_O_5_/ZnO_PVA = 1:1 caused partial hemoglobin release from erythrocytes into the supernatant. It was also found that the degree of hemolysis depends on the number of erythrocytes in the erythrocyte suspension. The most pronounced hemolysis was observed at an erythrocyte concentration of 1.0 ×106 thousand/mL. Therefore, this concentration of erythrocytes was used in further studies.

The method of cytocompatibility control, tested in this work, successfully proved to be a reliable method for controlling the general cytotoxicity of materials. Samples TiO_2_–SiO_2_–P_2_O_5_/CaO_PVA and Tokem-250_PVA, TiO_2_–SiO_2_–P_2_O_5_/La_2_O_3__PVA, and TiO_2_–SiO_2_–P_2_O_5_/La_2_O_3_:TiO_2_–SiO_2_–P_2_O_5_/CaO_PVA = 1:1 did not exceed the permissible levels of hemolysis for medical materials in contact with blood according to GOST ISO 10993-4-2020.

## 4 Conclusions

The spherical composites of TiO_2_–SiO_2_–P_2_O_5_/CaO, TiO_2_–SiO_2_–P_2_O_5_/La_2_O_3_, and TiO_2_–SiO_2_–P_2_O_5_/ZnO deposited on Tokem-250 were synthesized. The macroporous cation exchanger Tokem-250 has high selectivity to Ca^2+^, Zn^2+^, and La^3+^ ions, and it is a promising material for the creation of biomaterials. The composite structure was represented by the TiO_2_–SiO_2_–P_2_O_5_ film, and the inner part was filled with Ca^2+^/Zn^2+^/La^3+^ ions. The materials had a homogeneous structure with a uniform distribution of elements on the surface and high porosity (total pore volume was 0.48 cm^3^/g, average pore size was 15–26 nm), which is favorable for practical application. The effect of lanthanum and zinc additives on the biological properties of materials was studied. The active centers (Si^4+^, Ti^4+^) that are present on the surface of the obtained spherical composites promote the precipitation and mineralization of calcium phosphates on the surface of the materials in biological media. It was found that the accumulation of Ca^2+^ and Mg^2+^ ions proceeded fast on the surface of the TiO_2_–SiO_2_–P_2_O_5_/ZnO sample in the SBF (simulated body fluid) model solution, indicating a higher reacting capacity. In addition, the amount of calcium and phosphorus ions on the surface of the TiO_2_–SiO_2_–P_2_O_5_/La_2_O_3_ sample was higher due to the ability of lanthanum to coordinate a large number of ions (CN = 10). It was found that the introduction of a binder additive slightly reduced the formation of an apatite-like layer on the surface of samples at the first stage.

The presence of zinc ions in the system caused a partial release of hemoglobin from erythrocytes into the supernatant. The samples with lanthanum ions reduced the amount of protein in plasma after incubation, which is a good candidate for practical application. However, due to the presence of the strong antibacterial effect of zinc oxide, it should be used as a modifying additive in other systems to enhance the bioactive and functional properties.

The advantages of the obtained biomaterials based on the Tokem-250 cationite over other classes of materials are ease of manufacturing, simple recycling, high bioproperties, specified shape, and the porosity of the surface. Polymer frameworks promote the formation of new bone tissue, after which the framework breaks down into simple substances and is excreted from the body. Synthetic polymers are more preferred materials for regenerative medicine than natural polymers because they are easier to process and can be customized to produce a wider range of mechanical properties. Metal implants have been of great clinical importance in the medical field for a long time. However, in recent years, research has been focused on the development of various biomedical composite materials because they can be excellent alternatives to replacement tissues.
